# Cerebral blood flow-oxygen metabolism uncoupling at 24 hours after stroke thrombectomy

**DOI:** 10.1177/0271678X261501263

**Published:** 2026-10-20

**Authors:** Carolyn A Lomahan, Georgios Kalarakis, Marie L Luby, Staffan Holmin, Johan Lundberg, Michael V Mazya, Lawrence L Latour, Fabian Arnberg-Sandor

**Affiliations:** 1 Department of Clinical Neuroscience, Karolinska Institutet, Stockholm, Sweden; 2 Acute Cerebrovascular Diagnostics Unit, Stroke Branch, NINDS/NIH, Bethesda, MD, USA; 3 Department of Neuroradiology, Karolinska University Hospital, Stockholm, Sweden; 4 Department of Neurology, Karolinska University Hospital, Stockholm, Sweden

**Keywords:** Acute ischemic stroke, cerebral hemodynamics, endovascular therapy, magnetic resonance imaging, oxygen

## Abstract

**Background:**

Following successful endovascular therapy (EVT) for acute ischemic stroke, tissue with abnormal perfusion may have impaired oxygen extraction and metabolism. MRI model-based oxygen extraction fraction (OEF_max_) and cerebral metabolic rate of oxygen (CMRO_2_) were characterized in hypoperfusion and hyperemia regions 24 h post-EVT.

**Methods:**

Eighty-four patients were included with anterior circulation vessel occlusion, successful recanalization, and an MRI at 24 h post-EVT. Time-to-maximum (T_max_) and cerebral blood flow (CBF) were evaluated for hypoperfusion and hyperemia. CBF, OEF_max_, and CMRO_2_ were quantified using (i) segmentation-based and (ii) single slice region of interest (ROI) analyses. Normalized CBF, OEF_max_, and CMRO_2_ were calculated by dividing the segmentation or ROI value by the contralateral value.

**Results:**

Sixty-seven (80%) patients had abnormal perfusion; hypoperfusion in 22 (26%), hyperemia in 36 (43%), and both in 9 (11%). Compared to control, hypoperfused regions demonstrated a 62% CBF decrease, 52% CMRO_2_ decrease, and 68% OEF_max_ increase (*p* < 0.01). Hyperemic regions demonstrated a 58% CBF increase, 26% CMRO_2_ increase, and 25% OEF_max_ decrease (*p* < 0.01). Hyperemia had no significant correlation between normalized CBF and CMRO_2_ (*p* = 0.14).

**Conclusion:**

Hyperemia regions demonstrated cerebral blood flow-oxygen metabolism uncoupling, suggesting the underlying mechanism as loss of cerebrovascular autoregulation rather than hypermetabolic demand.

## Graphical abstract

**Graphical Abstract:**
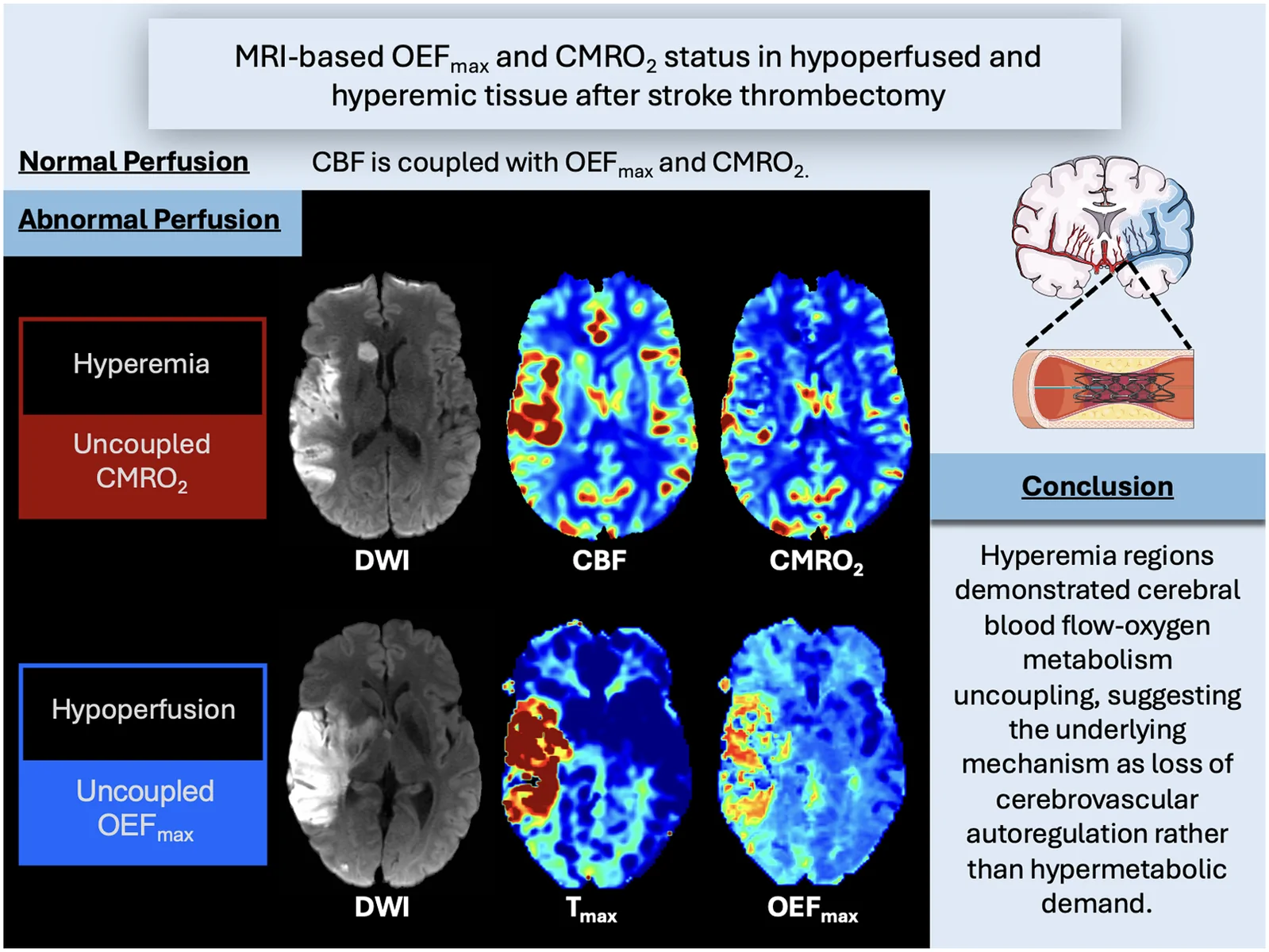
This is a visual representation of the abstract.

## Introduction

Patients with acute ischemic stroke (AIS) treated with endovascular therapy (EVT) may exhibit persistent or newly developed abnormal tissue perfusion, appearing as hypoperfusion or hyperemia, even after successful recanalization.^1,2^ Maximum oxygen extraction fraction (OEF_max_) and cerebral metabolic rate of oxygen (CMRO_2_) in the abnormally perfused regions may provide insight into underlying metabolic tissue changes after cerebral reperfusion and mechanistic causes of hyperemia. Residual perfusion disturbances are observed in approximately half of EVT-treated patients, and are associated with worse clinical outcomes, emphasizing the need to better characterize physiological parameters of tissue viability and subsequent clinical impact.^3–5^

In successfully recanalized patients, abnormal perfusion may entail hypoperfusion, hyperemia, or both at 24 h post-EVT. Hypoperfusion is an abnormal decrease in cerebral blood flow with time-to-maximum (T_max_) delay >6 s, that may have not been averted after reperfusion or may occur as a manifestation of distal emboli or vasospasm.^6,7^ Hyperemia, or an abnormal increase in cerebral blood flow, is likely the result of a loss of cerebrovascular autoregulation or a compensation for increased tissue metabolic demand.^8^

Recanalization of the occluded large or medium vessel alone may not be sufficient for maximal tissue salvage. Given the close relationship between blood flow and oxygen parameters, OEF_max_ and CMRO_2_ must also not be impaired. OEF_max_ represents the proportion of oxygen extracted from the blood into the tissue and CMRO_2_ is the rate at which brain tissue consumes oxygen. In the post-EVT setting, OEF_max_ has been explored in relation to penumbra detection, infarct prediction, and changes through the acute, subacute, and chronic phases.^9–11^ CMRO_2_ in the infarct is elevated in the acute phase, then normalizes.^12^ For advanced perfusion maps, the majority of the body of research has been performed with OEF_max_, with questions remaining about CMRO_2_ in stroke following reperfusion.

While positron emission tomography (PET) is considered the gold standard for measuring OEF_max_, it has limited clinical applicability in the acute setting due to the need of a cyclotron and complex procedures to administer ^15^O radiotracers.^13,14^ Recent advances in MRI dynamic susceptibility contrast (DSC) perfusion weighed imaging (PWI) post-processing have enabled the generation of model-based oxygen perfusion maps, OEF_max_ and CMRO_2_, in AIS.^10,15,16^ OEF_max_ measurements from the DSC-MRI and ^15^O PET approaches had moderate correlation.^17^ The MRI model-based oxygen perfusion maps may be utilized to provide additional information about the metabolic status of tissue following reperfusion, which could present an opportunity to develop therapeutic interventions for individual patients. This is an attractive approach to investigate the status of oxygen parameters in brain tissue regions experiencing hypoperfusion or hyperemia.

The study aim was to characterize MRI model-based OEF_max_ and model-based CMRO_2_ in areas of hypoperfusion or hyperemia at 24 h post-EVT. Revealing oxygen parameter status may provide evidence to the underlying physiological mechanisms of hypoperfusion and hyperemia.

## Material and methods

### Ethical considerations

This retrospective, observational study protocol was reviewed and approved by the Swedish ethical review authority, approval number 2024-02970-01, and performed in accordance with the ethical principles set forth by the Declaration of Helsinki.

### Consent to participate

Informed consent to participate was waived by the Swedish ethical review authority.

### Study population

This is a retrospective analysis of patients presenting to a single center, Karolinska University Hospital (Stockholm, Sweden). The study included adult patients with (i) anterior circulation large or medium vessel occlusion, (ii) EVT attempted, (iii) follow-up MRI obtained at 24 h post-EVT, including post-processed DSC PWI and diffusion weighted imaging (DWI), and (iv) achieved successful reperfusion based on the modified thrombolysis in cerebral infarction (mTICI) score of 2b, 2c, or 3. Patients were included if they presented between October 2024 and October 2025. During this time, Karolinska University Hospital piloted the implementation of MRI based follow-up imaging for EVT patients, rather than CT, given the superior sensitivity and specificity, safety, and efficacy previously demonstrated by global centers.^4,18,19^ Therefore, an MRI was initially obtained in patients who presented when an MRI technologist was available. Clinical information including comorbidities, medications at admission, prestroke modified Rankin Scale (mRS), admission National Institutes of Health Stroke Scale (NIHSS), thrombolysis administration, EVT target vessel location, mTICI score, 24 h NIHSS, and 90-day mRS were obtained from the local database and electronic health records. Early neurologic improvement (ENI) was defined as a decrease in NIHSS score of ⩾4 points from admission to 24 h, or a total score of 0–1 at 24 h.

### Imaging protocol

Follow-up MRI was obtained on a Signa Premier 3T scanner (GE Healthcare, WI, USA) at 24 h (±6 h) after recanalization. The MR imaging protocol included diffusion tensor imaging (DTI), susceptibility weighted imaging (SWI), fluid attenuated inversion recovery (FLAIR), and DSC perfusion-weighted imaging. The DWI protocol parameters were TR/TE: 4902 ms/ 66 ms, *b* = 0 and *b* = 1000 s/mm^2^, isotropically weighted, and a 192/192 acquisition matrix. The DSC PWI had the following parameters: TR/TE: 1500 ms/22 ms and a 128 × 128 matrix. During acquisition, a bolus of gadolinium contrast was administered with a power injector. MRI DSC perfusion maps, including the model-based oxygen sequences, were generated with Cercare Medical NeuroSuite (Cercare Medical, Denmark). The arterial input function was autonomously identified and used to derive the perfusion maps with 40 axial slices, each 4mm thick. Perfusion maps generated include CBF, T_max_, OEF_max_, and CMRO_2_. The software post-processing computation methods are described and utilized in previously published studies.^16,20–22^

In short, the processing pipeline models oxygen extraction capacity as a function of tissue oxygen tension and capillary transit time characteristics in steady state, where $CMRO_{2}=CBF\;\times \;C_{A}\times OEF_{max}$ ,^15,23^ and C_A_ is arterial oxygen concentration. The model assumptions of steady state include constant arterial oxygen, oxygen transport limited by diffusion from capillaries to surrounding tissue, and notably, independent capillary flow. Therefore, the model acknowledges that blood may flow at different speeds in different capillaries, rather than homogeneous flow assumed in traditional models. CMRO_2_ is defined as the oxygen metabolism that can be supported by the vasculature assuming that metabolic rate cannot surpass the oxygen that diffuses from the blood to tissue, given the capillary architecture and cerebral blood flow. OEF_max_ is determined by both blood flow and capillary transit time heterogeneity.

### Qualitative image analysis

Each case was read by two of three raters (CAL and GK or FA) to evaluate the perfusion maps for the presence or absence of hypoperfusion (Figure 1(a)) and/or hyperemia (Figure 1(b)) at 24 h post-EVT. The affected vascular territory hemisphere was assessed for the presence or absence of perfusion abnormalities. In cases of disagreement, perfusion status was assessed by an additional consensus reading with two raters evaluating the perfusion imaging together.

**Figure 1.**
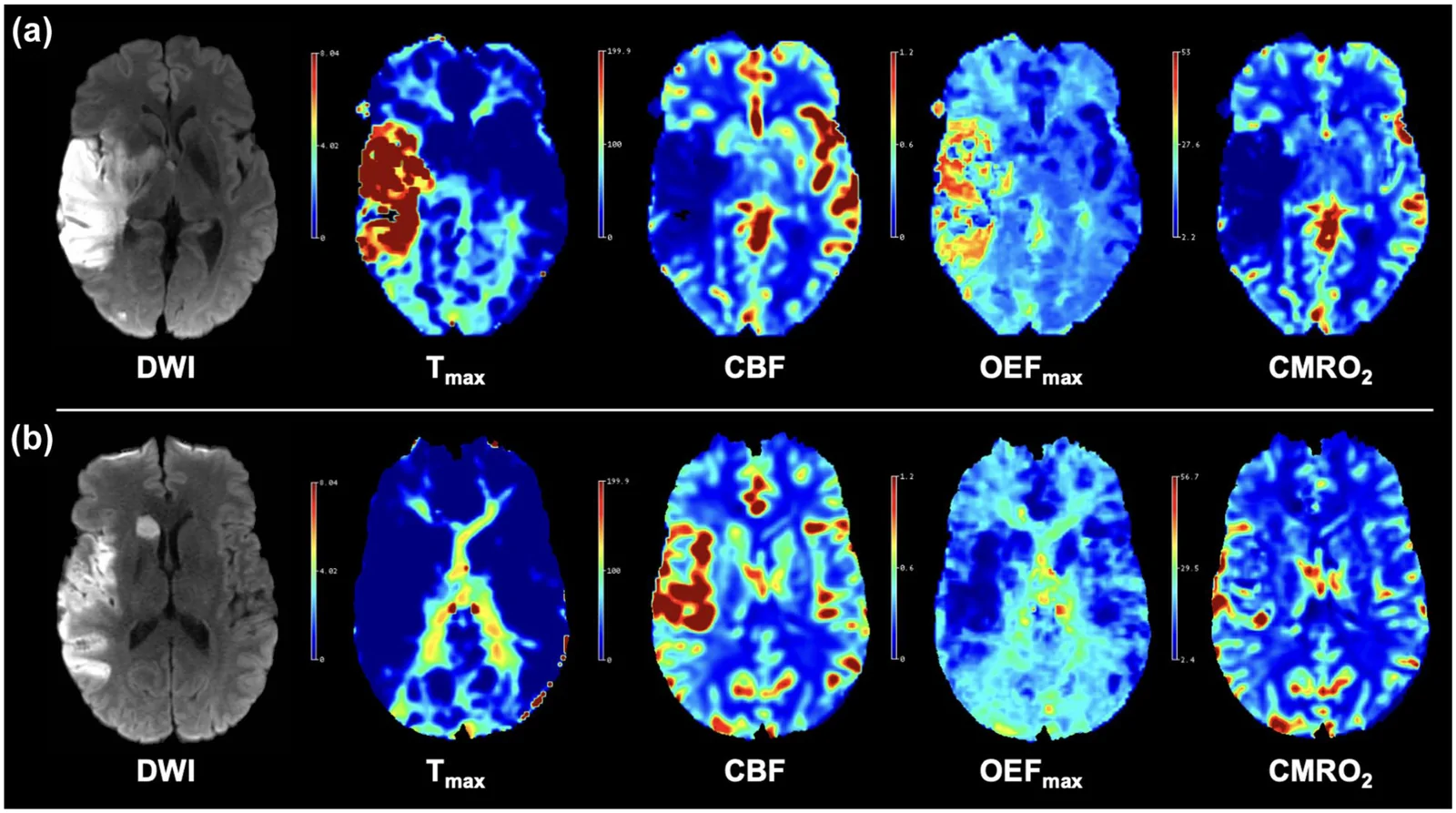
The DWI lesion and regions of hypoperfusion and hyperemia can be appreciated on perfusion sequences including T_max_, CBF, model-based OEF_max_, and model-based CMRO_2_ at 24 h post-endovascular therapy. (a) Hypoperfusion was detected in the right MCA vascular territory for a 55-year-old with an admit NIHSS of 13 who underwent successful revascularization (mTICI 2b). (b) Hyperemia was detected in the right MCA vascular territory for a 57-year-old with an admit NIHSS of 14 who underwent successful revascularization (mTICI 2c).

Evaluation for regions of hypoperfusion and hyperemia was performed using qualitative visual assessment supplemented by quantitative analysis. Hypoperfusion was visually defined as a conspicuous increase in T_max_ or decrease in CBF and quantitatively defined as regions with corresponding delay on T_max_>6 s. These hypoperfused areas were interpreted to contain tissue with macrovascular hypoperfusion, rather than microvasculature hypoperfusion due to the no-reflow phenomenon in capillaries. Hyperemia was visually defined as a conspicuous increase in CBF with quantitative support by cerebral blood flow increases in hyperemic regions compared to a homologous contralateral control regions.

### Quantitative image analysis

#### Hypoperfusion, hyperemia, and DWI volumes

Volumes (mL) at 24 h were calculated for regions of hypoperfusion on T_max_, hyperemia on CBF, and lesion on DWI by a single rater (CAL). The hypoperfusion volumes were obtained in Mipav (v11.2.0, Center for Information Technology, NIH) by manually selecting the affected vascular territory hemisphere and applying a threshold of T_max_>6 s.^24^ Visually guided hyperemia volumes were measured from CBF by creating a segmentation on a slice-by-slice basis with a validated semi-automated method in Mipav.^8^ The DWI was segmented on a slice-by-slice basis with a similar validated semi-automated method, and corresponding volumes were automatically calculated using Mipav.^24^

#### Model-based OEF_max_ and CMRO_2_ quantification

Two analyses were performed to quantify model-based OEF_max_ and CMRO_2_ in regions of hypoperfusion (Figure 2(a)) and hyperemia (Figure 2(b)) based on: (i) segmentations for hypoperfusion and hyperemia and (ii) single-slice regions of interest (ROI) selected in the most visually conspicuous slice of abnormal perfusion. Normal perfusion was evaluated in patients without perfusion abnormalities in the vascular territory EVT was performed with the single-slice ROI analysis (Figure 2(c)). Single-slice ROIs were chosen to minimize the dilution of physiologically distinct regions, while volumetric analysis provided complementary information.

**Figure 2.**
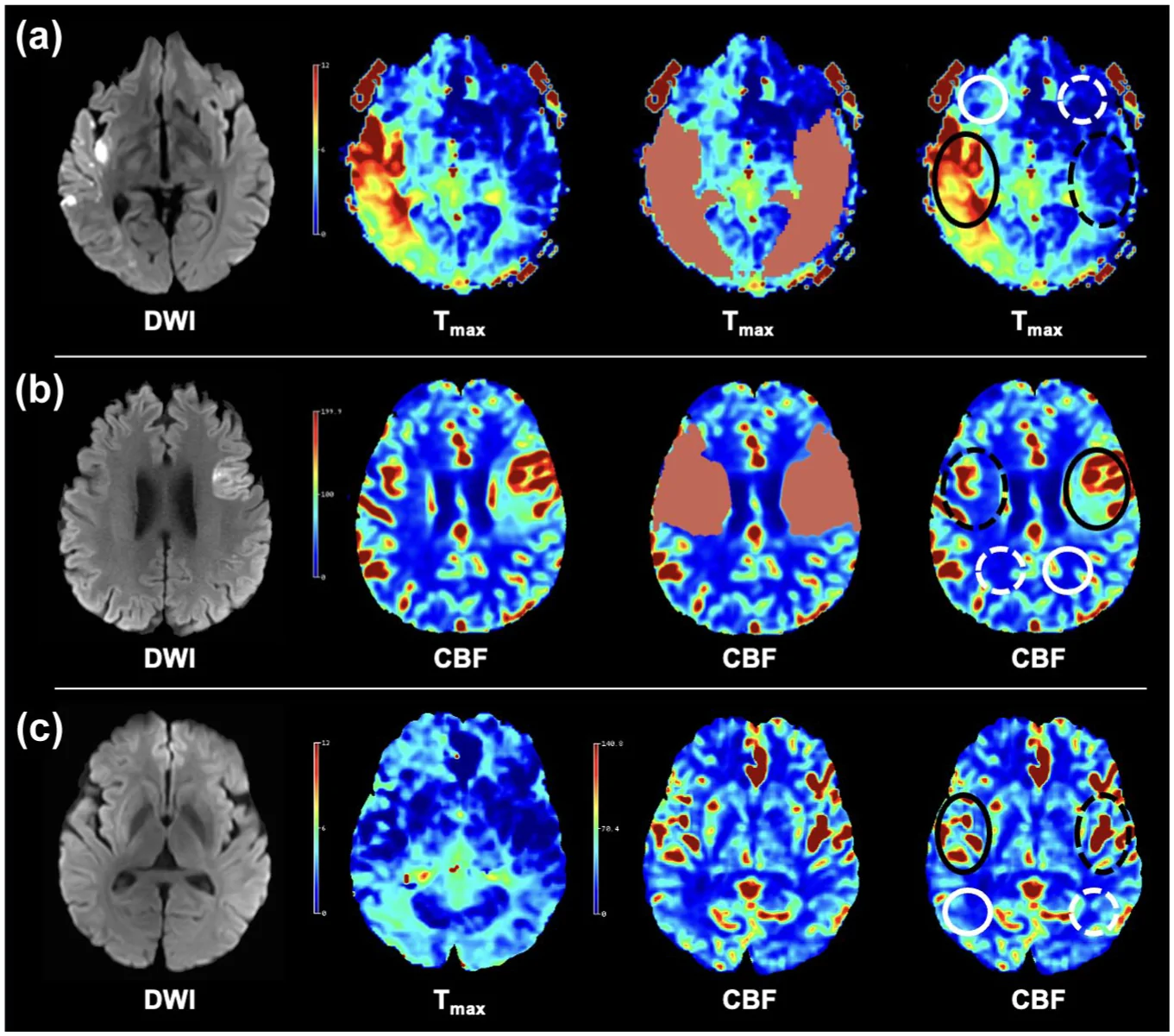
Quantification of model-based OEF_max_ and CMRO_2_ was performed using two methods: segmentation-based and single-slice ROI based. Normalized CBF, OEF_max_, and CMRO_2_ were calculated by dividing the value of the segmentation or ROI by the homologous contralateral control value. The first column is the corresponding DWI slice. (a) Hypoperfusion is shown on T_max_ maps: without annotation (second column), with segmentation of the affected and contralateral regions (third column), and with a manual ROI placed in the most visually conspicuous hypoperfused region (black), a contralateral ROI (dotted black), and an unaffected control region (white/dotted white; fourth column). (b) Hyperemia is shown on CBF using the same display format: unannotated (second column), segmented (third column), and with manual ROI (fourth column). (c) Normal perfusion is shown on T_max_ (second column) and CBF (third column). CBF has a manual ROI (black) in the treated vascular territory, the contralateral side (dotted black), and control regions (white/dotted white; fourth column).

I. Segmentations and contralateral homologous mirror segmentations were calculated in Mipav, with visual confirmation of mirror segmentations encompassing brain tissue. The segmentation of hypoperfusion or hyperemia and a mirror segmentation were applied to CBF, OEF_max_, and CMRO_2_ perfusion maps in 3D Slicer.^25^ In patients with both perfusion abnormalities, each segmentation was applied separately to the perfusion maps in different sessions. The mean and standard deviation of the value in the segmentations were obtained in 3D Slicer. Normalized CBF, OEF_max_, and CMRO_2_ were calculated by the segmentation of interest value divided by the homologous contralateral segmentation value.

II. The single-slice ROI method was performed in the Cercare Medical Neurosuite software (Cercare Medical, Denmark). For patients with visually confirmed presence of hypoperfusion and/or hyperemia at 24 h, a ROI was placed in the most representative region of perfusion abnormality with a corresponding homologous contralateral ROI on a single slice of the CBF. On the same slice, a control ROI and a homologous contralateral ROI were applied in an area of tissue without a perfusion abnormality. Control ROIs were selected in areas of tissue that avoided major arteries and abnormal perfusion areas. The ROI was normalized by dividing the ROI value by the contralateral ROI value. In patients with no perfusion abnormality at 24 h, normalized CBF, OEF_max_, and CMRO_2_ were calculated by using ROIs in the affected vascular territory hemisphere on the side of EVT and a homologous contralateral control region.

### Correlation analysis

For patients with hypoperfusion and hyperemia, segmentation-based normalized CBF versus normalized model-based OEF_max_ and normalized CBF versus model-based CMRO_2_ were analyzed for correlation using the Pearson correlation coefficient. The control normalized values were utilized from the single-slice ROI method.

### Statistical analysis

Clinical and imaging data were reported as frequencies (sample size and percentage), or median (interquartile range (IQR 25–75)) as appropriate. Patients were grouped by hypoperfusion, hyperemia, both, or normal perfusion at 24 h. Normalized CBF, OEF_max_, and CMRO_2_ for hypoperfusion or hyperemia and control were compared using the Wilcoxon signed rank sum test for paired data. Variables were compared using the Wilcoxon signed rank sum test or Kruskal-Wallis test as appropriate for continuous variables and Chi-square for categorical variables. The association between perfusion abnormality groups and 24 h NIHSS was evaluated using linear regression models. Univariate analysis and a multivariable linear regression model were performed and incorporated variables that were significant at the *p* < 0.10 level in the univariate model. Regression coefficients (β) with 95% confidence intervals (CI) and *p*-values were reported. The Dice coefficient was calculated between DWI lesion masks and hyperemia and hypoperfusion masks at 24 h. 3D Slicer BRAINS registration module with 6 degrees of freedom was used to co-register the CBF, T_max_, and masks to the DWI. Interrater agreement was calculated with kappa for qualitative perfusion abnormality reads between the readers (CAL, GK, and FA). The Pearson correlation coefficient was reported for the normalized CBF versus OEF_max_ and CBF versus CMRO_2_. Coefficients were considered weak between 0.10 and 0.29, moderate between 0.3 and 0.49, and strong between 0.5 and 1. All statistical and visual data analyses were performed using RStudio (version 2024.09.1+394).

## Results

### Clinical and imaging characteristics

Of 152 patients with an MRI at 24 h post-EVT, 84 were included in the analysis. Patients were excluded due to posterior circulation stroke (*n* = 41), no DSC PWI obtained (*n* = 18), post-EVT mTICI <2b (*n* = 5), and MR timing falling outside the window of 24 h (± 6 h; *n* = 4; Supplemental Figure I). Patients had a median age of 70 years, 36 (43%) female, and 44 (52%) had a M1 occlusion (Table 1). There were no differences in comorbidities or medications on admission between groups based on perfusion status at 24 h. For all patients, the mTICI was 2b for 29 (35%) patients, 2c for 16 (19%), and 3 for 39 (46%). The groups had a significant difference in mTICI scores (*p* = 0.007). The 24 h NIHSS, ENI, and 24 h DWI lesion volume were significantly different between patients based on perfusion status (*p* = 0.001, *p* < 0.001, *p* = 0.001, respectively; Table 1). There was no statistical significance between rates of thrombolysis, admission NIHSS, admission to thrombectomy start time, thrombectomy vessel location, and 90-day mRS for patients based on perfusion status.

**Table 1.** Patient clinical characteristics, treatment characteristics, clinical, and imaging findings for study patients and cohorts stratified by perfusion status at 24 h.

Variable median (IQR); *n*(%)	All patients (*n* = 84)	Hypoperfusion (*n* = 22)	Normal (*n* = 17)	Hyperemia (*n* = 36)	Both (*n* = 9)	*p*-Value
Patient clinical characteristics						
Age (years)	70 (58–78)	69 (62–76)	76 (61–79)	70 (58–77)	74 (45–82)	0.743
Sex (female) (%)	36 (43)	10 (45)	6 (35)	15 (42)	5 (56)	0.781
Risk factor (%)						
Atrial fibrillation	21 (25)	3 (14)	4 (24)	11 (31)	3 (33)	0.465
Diabetes	11 (13)	4 (18)	2 (12)	4 (11)	1 (11)	0.874
Hypertension	49 (58)	12 (55)	10 (59)	21 (58)	6 (67)	0.964
Previous stroke	9 (11)	1 (4.5)	2 (12)	5 (14)	1 (11)	0.779
Smoking	10 (12)	2 (9.1)	2 (12)	4 (11)	2 (22)	0.781
Medications at admission (%)						
Aspirin	14 (17)	4 (18)	2 (12)	7 (19)	1 (11)	0.948
Clopidogrel	2 (2.4)	0 (0)	0 (0)	1 (2.8)	1 (11)	0.309
Apixaban	15 (18)	2 (9.1)	3 (18)	8 (22)	2 (22)	0.65
Treatment characteristics, clinical, and imaging findings						
Prestroke mRS	0 (0–0)	0 (0–0)	0 (0–0)	0 (0–0)	0 (0–3)	0.824
Admission NIHSS	12 (6–17)	10 (4–14)	10 (9–15)	12 (6–18)	14 (6–17)	0.728
Thrombolysis (%)	25 (30)	5 (23)	7 (41)	12 (33)	1 (11)	0.385
Admission to thrombectomy start time (min)	282 (105–635)	499 (247–750)	269 (110–615)	331 (132–790)	107 (91–342)	0.112
Thrombectomy vessel location (%)						0.474
ICA	17 (20)	7 (32)	3 (18)	6 (17)	1 (11)	
M1	44 (52)	10 (45)	8 (47)	20 (56)	6 (67)	
M2	22 (26)	5 (23)	6 (35)	10 (28)	1 (11)	
M3	1 (1)	0 (0)	0 (0)	0 (0)	1 (11)	
mTICI score (%)						**0.007**
2b	29 (35)	13 (59)	3 (18)	8 (22)	5 (56)	
2c	16 (19)	3 (14)	1 (5.9)	10 (28)	2 (22)	
3	39 (46)	6 (27)	13 (76)	18 (50)	2 (22)	
24 h NIHSS	2 (0–7)	6 (4–14)	0 (0–2)	1 (0–4)	2 (2–16)	**0.001**
ENI (%)	58 (69)	4 (18)	17 (100)	32 (89)	5 (56)	**<0.001**
24 h DWI Lesion Volume (mL)	11 (3–31)	21 (11–52)	3 (1–5)	10 (2–26)	23 (12–48)	**0.001**
90-day mRS*	2 (0–3)	3 (1–4)	2 (0–3)	1 (0–3)	1 (1–3)	0.154

* Missing 1 patient due to moving out of the country.

*p* < 0.05 bolded.

Univariate linear regression evaluating perfusion abnormality groups and 24 h NIHSS identified admission NIHSS, thrombolysis, EVT target vessel, and mTICI score as significant predictors (Table 2). Compared to no perfusion abnormality, patients with hypoperfusion and both were associated with worse 24 h NIHSS, but not hyperemia. A multivariate model adjusting for the significant variables from the univariate analysis demonstrated hypoperfusion and both remained significant. However, EVT target vessel was no longer independently associated with 24 h NIHSS. Separate models adjusting for each variable individually also demonstrated hypoperfusion and both had significantly higher 24 h NIHSS (Supplemental Table II). Admission NIHSS and EVT target vessel remained independently associated with 24 h NIHSS, whereas thrombolysis and mTICI did not.

**Table 2.** Univariate and multivariable linear regression analyses evaluating the association between perfusion abnormality groups and 24 h NIHSS.

Variable	Univariate		Multivariate Model	
	β (95% CI)	*p*-Value	Adjusted β (95% CI)	*p*-Value
Perfusion abnormality groups				
Hyperemia	1.87 (−2.21–5.94)	0.4	0.76 (−2.60–4.13)	0.652
Hypoperfusion	8.04 (3.57–12.51)	**<0.001**	8.04 (4.15–11.94)	**<0.001**
Both	7.48 (1.77–13.18)	**0.011**	6.51 (1.40–11.62)	**0.013**
Age	0 (−0.10–0.09)	>0.9		
Sex (female)	−0.55 (−3.89–2.79)	0.7		
Admission NIHSS	0.50 (0.31–0.70)	**<0.001**	0.53 (0.34–0.73)	**<0.001**
Preadmission mRS score	0.22 (−1.16–1.61)	0.7		
Thrombolysis	−3.59 (−7.12–0.05)	**0.047**	−2.29 (−4.99–0.42)	**0.096**
Admission to thrombectomy start time (min)	0 (0–0.01)	0.2		
EVT target vessel				
M1	−5.39 (−9.50–−1.27)	**0.011**	−1.65 (−5.20–1.90)	0.357
M2	−7.50 (−12.15–−2.85)	**0.002**	−0.28 (−4.53–3.98)	0.897
M3	−8.00 (−22.82–6.82)	0.3	−8.17 (−21.00–4.66)	0.209
mTICI score				
2b	3.70 (0.07–7.33)	**0.046**	0.46 (−2.61–3.52)	0.768
2c	3.75 (−0.64–8.14)	**0.093**	3.99 (0.45–7.53)	**0.028**

p < 0.10 bolded.

### Qualitative image analysis

Persistent abnormal perfusion at 24 h post-EVT was detected in 67 (80%) patients; hypoperfusion in 22 (26%), hyperemia in 36 (43%), and both in 9 (11%). The interrater agreement for the presence or absence of hypoperfusion and/or hyperemia among three raters was strong, with κ = 0.771 (CAL and FA) and κ = 0.875 (CAL and GK). During the initial independent read, 70/84 (83%) cases had concordant reads, and 14/84 (17%) had discordant reads that underwent consensus reads.

### Quantitative image analysis

#### Hypoperfusion, hyperemia, and DWI volumes

The volume for hypoperfusion was 7 mL (IQR, 1–11) and for hyperemia was 37 mL (IQR, 12–68). Abnormal perfusion status was associated with significantly larger DWI lesion volume compared to patients with normal perfusion (*p* = 0.001; Table 1). The Dice coefficient was 0.16 (0.01–0.32) for DWI lesion and hyperemia masks and was 0.02 (0–0.12) for DWI lesion and hypoperfusion masks.

#### Model-based OEF_max_ and CMRO_2_ quantification

There was a significant difference between segmentation-based normalized CBF, model-based OEF_max_ and CMRO_2_ based on perfusion status (*p* < 0.01; Figure 3). Compared to control, hypoperfused regions demonstrated a 62% CBF decrease, 52% CMRO_2_ decrease, and 68% OEF_max_ increase (*p* < 0.01). Hyperemic regions demonstrated a 58% CBF increase, 26% CMRO_2_ increase, and 25% OEF_max_ decrease (*p* < 0.01). The single-slice ROI normalized ratio had statistically significant differences compared to control in the same direction but different magnitudes (Supplemental Table I). The control normalized CBF, OEF_max_, and CMRO_2_ were ~1.00 across perfusion maps for hypoperfusion and hyperemia, as expected for the control region (Supplemental Table I).

**Figure 3.**
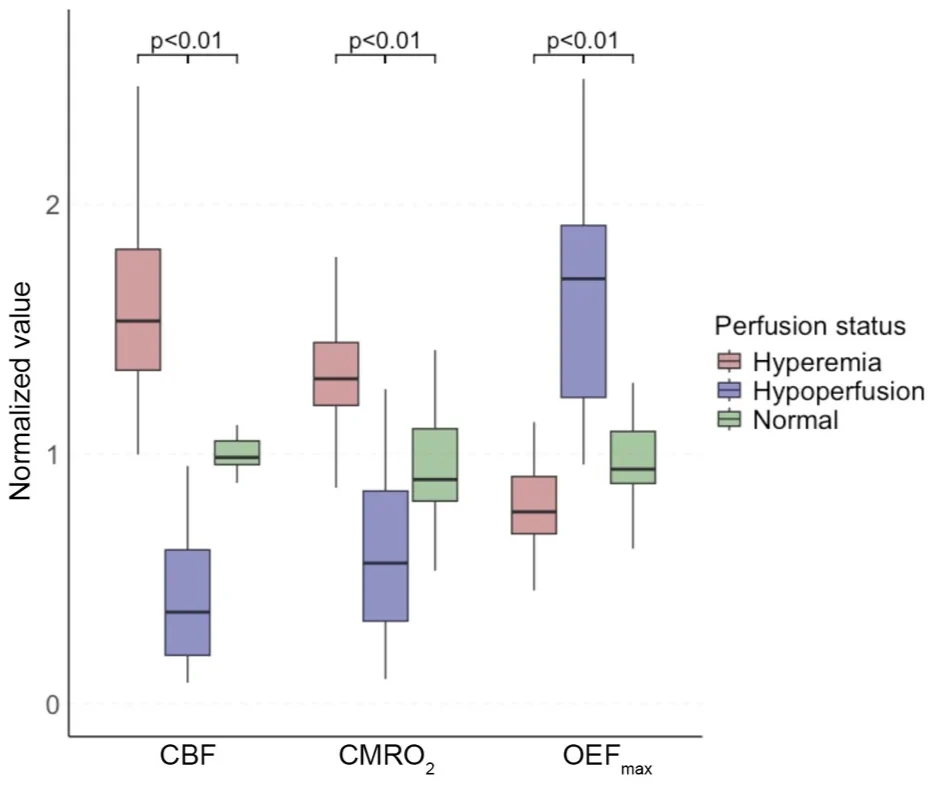
Segmentation-based normalized CBF, model-based CMRO_2_, and model-based OEF_max_ for patients with hypoperfusion, hyperemia, and single-slice ROI based normal perfusion.

### Correlation analysis

Segmentation-based normalized CBF versus normalized model-based OEF_max_ and model-based CMRO_2_ for patients with hypoperfusion, hyperemia, and single-slice based control regions were analyzed for correlation. For hypoperfusion, the normalized CBF versus OEF_max_ was not statistically significant (*p* = 0.16; Figure 4(a)). However, a significant correlation coefficient was demonstrated between normalized CBF versus CMRO_2_ (Pearson’s correlation coefficient *r* = 0.55, (95% CI, 0.22–0.77), *p* < 0.01; Figure 4(b)). For hyperemia, the normalized CBF versus OEF_max_ demonstrated a significant correlation (Pearson’s correlation coefficient *r* = −0.81, (95% CI, −0.89–−0.68), *p* < 0.01; Figure 4(c)). Notably, hyperemia did not have a significant correlation between normalized CBF versus CMRO_2_ (*p* = 0.14; Figure 4 (d)). For comparison, control regions demonstrated a significant correlation between normalized CBF versus OEF_max_ (Pearson’s correlation coefficient *r* = −0.38, (95% CI, −0.54–−0.20), *p* < 0.01; Figure 4(e)) and CBF versus CMRO_2_ (Pearson’s correlation coefficient *r* = 0.28, (95% CI, 0.08–0.46), *p* < 0.01; Figure 4(f)).

**Figure 4.**
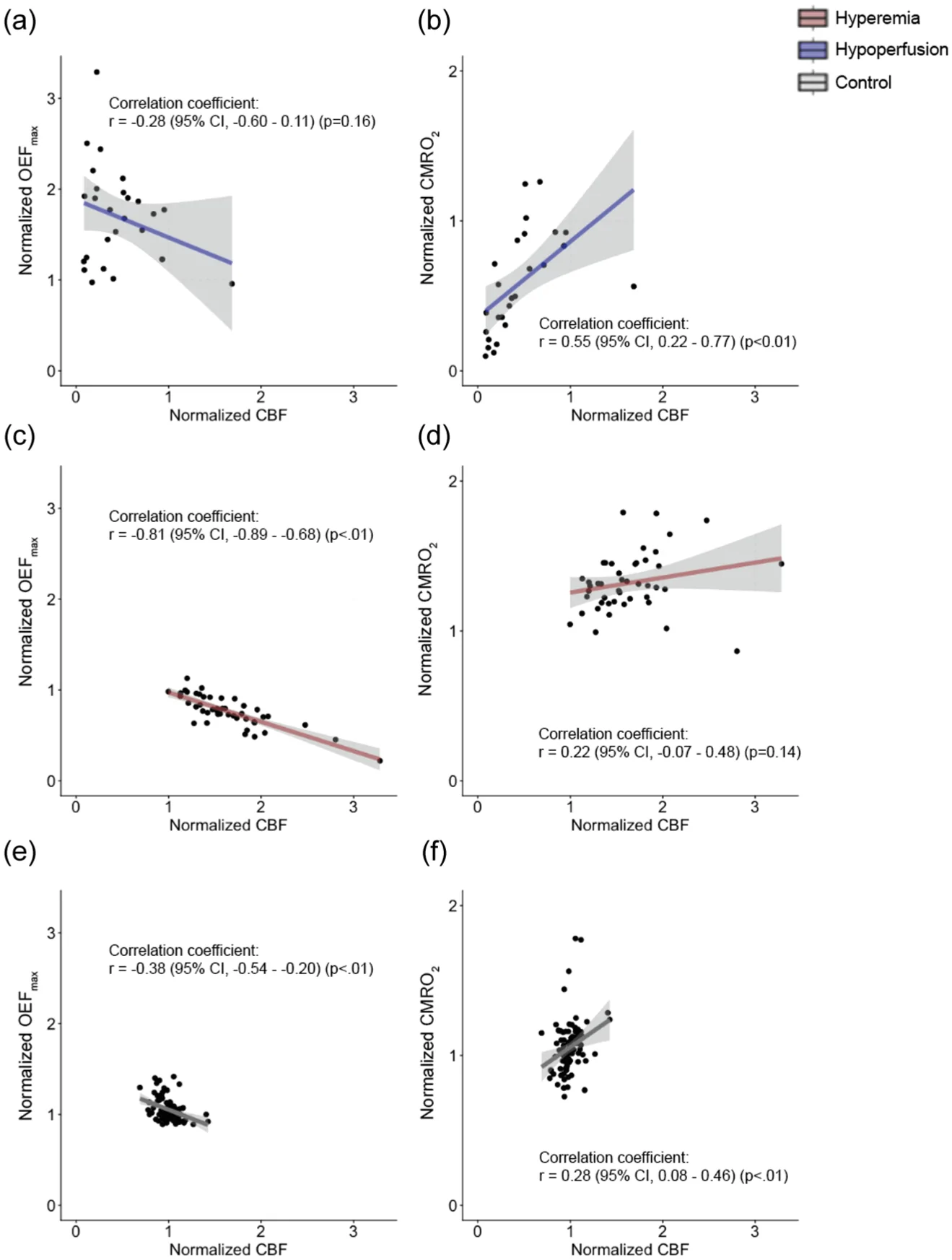
Plotted correlations between segmentation-based normalized CBF versus model-based OEF_max_ and CBF versus model-based CMRO_2_ for patients with hypoperfusion (a and b) and hyperemia (c and d). Single slice-based control normalized CBF versus model-based OEF_max_ and CBF versus model-based CMRO_2_ for all patients (e and f).

## Discussion

In this retrospective study, we characterized CBF, MRI model-based OEF_max_, and MRI model-based CMRO_2_ in hypoperfusion and hyperemia regions at 24 h after stroke thrombectomy. Hypoperfusion regions had a significant correlation between decreased normalized CBF and normalized CMRO_2_, reflecting the physiological correlate of less blood flow and less oxygen metabolism, whereas no significant correlation was found between normalized CBF and normalized OEF_max_. Hyperemia regions had a significant strong correlation between normalized CBF and normalized OEF_max_, in which tissue with increased blood flow had less OEF_max_. Notably, no significant correlation was found for hyperemia between increased normalized CBF and normalized CMRO_2_. This data suggests hypoperfused tissue had coupled reductions in blood flow and oxygen metabolism, whereas hyperemic tissue demonstrated cerebral blood flow-oxygen metabolism uncoupling at 24 h after thrombectomy.

Under normal blood flow dynamics, CBF, OEF_max_, and CMRO_2_ are coupled and co-vary. However, ischemic injury followed by abnormal reperfusion may result in these relationships uncoupling. Abnormal blood flow dynamics may additionally become altered based on oxygen availability in the blood, tissue demand for oxygen, changes to vascular integrity, or capillary transit time heterogeneity. Therefore, OEF_max_ and CMRO_2_ alterations may indicate failed metabolic compensation.

The physiological mechanism of hyperemia remains unclear, though it may result from loss of cerebrovascular autoregulation or hypermetabolism. Under normal physiological conditions, the homeostatic control of CBF is determined by the resistance of the vessels, which have cerebrovascular autoregulation: the response of the vessels to dilate or constrict to a range of CBF alterations.^26^ Surpassing the upper limit of autoregulation constriction may result in forceful dilation of the vessels, leading to uncontrolled excessive blood flow. Hyperemia has also been referred to as luxury perfusion syndrome, but conflicting studies have defined it as increased blood flow with or without a corresponding increase in cerebral metabolic rate of oxygen.^27–29^ Alternatively, flow increases may be driven by a hypermetabolic demand of oxygen in the tissue. Previous studies with functional magnetic resonance imaging (fMRI) based blood oxygenation level dependent (BOLD) signal changes demonstrates active neurons increase CMRO_2_, triggering an increase in CBF.^30^ In the setting of stroke post-EVT, our study findings showed hyperemia had CBF increases in excess to MRI model-based CMRO_2_ increases, similarly demonstrated with BOLD studies.^31,32^ Adding to this consistency with prior work, a novel finding emerges: hyperemic regions had no significant correlation between CBF and model-based CMRO_2_, suggesting region-specific cerebral blood flow-oxygen metabolism uncoupling. The lack of the tissue’s ability to extract oxygen from the blood due to vessels being maximally dilated may contribute to the lack of the association between CBF and CMRO_2_. This supports that the underlying mechanism of post-EVT hyperemia is more likely due to loss of cerebrovascular autoregulation, rather than hypermetabolic demand.

Our findings demonstrated 24 h post-EVT hypoperfused tissue exhibited increased model-based oxygen extraction and decreased metabolism, with a strong correlation coefficient found between decreased blood flow with less CMRO_2_. The lack of association with OEF_max_ may be due to severe blood flow reduction and oxygen demand reduction in parallel, reflecting hemodynamic compromise in which all available oxygen has been extracted to the tissue. Alternatively, the reduction in CMRO_2_ may drive the exceeded limit of a compensatory increase in OEF_max_ otherwise expected for hypoperfusion. A study with PET O^15^, looked at CMRO_2_ in perfusion-diffusion mismatch and similarly found a reduction of CMRO_2_ within 6 h from onset.^33^ The timing of oxygen parameters may become relevant if persistent hypoperfusion lasts beyond the subacute phase, as it may negate the clinical benefit of successful EVT.^34^ Furthermore, quantitatively defining hypoperfusion as T_max_>6 s represented macrovasculature hypoperfusion, rather than the no-reflow phenomenon in the microvasculature. Future directions may include investigating oxygen parameter alterations in regions of no-reflow to determine if this tissue has similar or different oxygen parameters to tissue with macrovasculature hypoperfusion.

Heterogeneous cerebral hemodynamics in the subacute phase of AIS treated with EVT intertwines cerebral blood flow, oxygen parameters, and tissue outcomes.^35,36^ Recent studies highlighted model-based OEF_max_ trends in relation to ischemic lesion recovery and penumbral tissue. An increase in OEF_max_ was associated with ischemic lesion recovery between admission and day 6.^16^ Our findings suggested low spatial similarity between DWI lesion and hyperemia and hypoperfusion at the 24 h timepoint, but the lesion may still be progressing, and improvement would be expected with a comparison to a final infarct volume at a week timeframe post-EVT. Preclinical and clinical studies have found that an increase in OEF_max_ was associated with the penumbra.^9,37^ While our study found that hypoperfusion regions had increased MRI model-based OEF_max_, a previous study utilizing CT perfusion found OEF_max_ values were lower in the penumbra of patients that achieved complete recanalization.^21^ The measurement of CBF, concomitant with evaluation of oxygen extraction and metabolism, has the potential to determine tissue progression to ischemia, as areas of abnormal perfusion were associated with significantly larger lesion volumes at 24 h compared to patients with normal perfusion.

The gold standard for OEF_max_ quantification is PET with ^15^O, but the application in a clinical setting is challenging. MRI-based OEF_max_ quantification techniques have been modeled in previous studies utilizing various techniques. OEF_max_ measurements between T_2_ relaxation under spin tagging (TRUST) MRI and quantitative blood level oxygenation dependent (BOLD) technique compared to PET showed no significant differences.^38,39^ Our study utilized DSC PWI post-processed maps which were found to have OEF_max_ measurements comparable to PET, which may have clinical implications.^17,23,40^

Despite advances in revascularization techniques, about half of ischemic stroke patients treated with EVT do not achieve a favorable clinical outcome.^41^ Abnormal perfusion may be a contributing factor, ranging from a residual perfusion deficit, futile recanalization, reperfusion injury, to hyperemia.^42–44^ Successful recanalization post-EVT is assessed based on the mTICI score but does not account for the temporal evolution of complex and dynamic blood flow changes. Thus, even with successful recanalization, an additional evaluation of cerebral blood flow may be warranted, as study findings demonstrated that a majority patients have heterogeneous perfusion status at 24 h. Furthermore, patients with abnormal perfusion were associated with poorer clinical outcomes, as indicated by significantly higher NIHSS at 24 h compared to patients with normal perfusion.

MRI follow-up at 24 h can serve as a timepoint for additional perfusion imaging to inform clinical decision-making and ultimately improve patient outcomes. Physiological disturbances on MRI may reflect secondary injury mechanisms not captured by NIHSS or mRS. Our study showed worse 24 h NIHSS in patients with abnormal versus normal perfusion, but no differences in the 90-day mRS. However, perfusion abnormalities may reflect secondary injury mechanisms not captured by global functional scales. Additional information from MRI may have significant clinical utility in characterizing secondary injury, like perfusion abnormalities. Adjunctive therapies may be offered as future interventions by selecting patients based on imaging targets that may gain additional benefit, rather than putting the patient at unnecessary risk. Oxygen parameters derived from DSC PWI may serve as a potential imaging target.

Our study has several limitations. First, previous studies demonstrated patients with mTICI 2b versus 3 have worse functional outcomes.^45,46^ Expectedly, patients with mTICI 2b had frequent hypoperfusion and were included in our study to further characterize the type of perfusion abnormalities within the group. These patients with hypoperfusion likely indicated presence of distal clots, rather than the no-reflow phenomenon observed in microvasculature. Second, the patient population may be less severe than those typically constituting large and medium vessel occlusion, due to the selection bias of including patients that obtained an MRI follow-up at 24 h. Third, the variable ROI size in the quantitative analysis was adjusted to capture a representative region of hypoperfusion or hyperemia on a single slice. However, the control size of the ROI, was appropriately consistent. Fourth, the number of patients in each perfusion status cohort is limited, and the multivariable analysis could only adjust for a limited number of clinical factors. Lastly, the study patient population is from a single center, and validation of the findings in a multicenter study is recommended.

In conclusion, our findings highlight an altered cerebral blood flow-oxygen parameter relationship in hypoperfusion and hyperemia regions at 24 h after successful recanalization in acute ischemic stroke patients. We demonstrated that regions with hyperemia had a strong correlation between increased cerebral blood flow and decreased OEF_max_, whereas hypoperfused regions had no significant correlation with OEF_max_. Hypoperfused regions had a coupled decrease in CBF and CMRO_2_. Hyperemia regions had cerebral blood flow-oxygen metabolism uncoupling, suggesting the underlying mechanism may be due to loss of cerebrovascular autoregulation rather than hypermetabolic demand.

## Supplemental Material

sj-docx-1-jcb-10.1177_0271678X261501263 – Supplemental material for Cerebral blood flow-oxygen metabolism uncoupling at 24 hours after stroke thrombectomy Supplemental material, sj-docx-1-jcb-10.1177_0271678X261501263 for Cerebral blood flow-oxygen metabolism uncoupling at 24 hours after stroke thrombectomy by Carolyn A Lomahan, Georgios Kalarakis, Marie L Luby, Staffan Holmin, Johan Lundberg, Michael V Mazya, Lawrence L Latour and Fabian Arnberg-Sandor in Journal of Cerebral Blood Flow & Metabolism

## Data Availability

All data were pseudo-anonymized and deidentified. Anonymized data can be made available upon reasonable request to the corresponding author, contingent on ethical authority approval (contact fabian.arnberg-sandor@regionstockholm.se).*
