# Supplementary material for: Cerebral blood flow-oxygen metabolism uncoupling at 24 hours after stroke thrombectomy
Source: J Cereb Blood Flow Metab. 2026 Oct 20:0271678X261501263. Online ahead of print. doi: 10.1177/0271678X261501263 (PMC13768318; doi:10.1177/0271678X261501263)
Supplement: Supplementary file 1 [file sj-docx-1-jcb-10.1177_0271678X261501263.docx]

Supplemental Figure I. Patient inclusion flowchart.

Supplemental Table I. Hypoperfusion and hyperemia segmentation-based and single slice-based control normalized ratio across perfusion maps.

| **Perfusion Status** | **Perfusion map** | **Segmentation normalized ratio** | **Single slice ROI normalized ratio** | **Single slice control normalized ratio** | **p-value** |
| --- | --- | --- | --- | --- | --- |
| Hypoperfusion  (n=31) | CBF | 0.37 (0.19 - 0.62) | 0.32 (0.16 - 0.59) | 0.99 (0.93 - 1.06) | **<0.001** |
|  | OEF_max_ | 1.73 (1.23 - 1.94) | 1.56 (1.33 - 2.13) | 1.05 (1.00 - 1.20) | **<0.001** |
|  | CMRO_2_ | 0.56 (0.33 - 0.85) | 0.59 (0.35 - 0.86) | 1.08 (0.96 - 1.16) | **<0.001** |
| Hyperemia  (n=45) | CBF | 1.56 (1.34 - 1.84) | 2.16 (1.66 - 3.14) | 0.98 (0.93 - 1.08) | **<0.001** |
|  | OEF_max_ | 0.77 (0.68 - 0.91) | 0.64 (0.50 - 0.75) | 1.02 (0.98 - 1.06) | **<0.001** |
|  | CMRO_2_ | 1.30 (1.20 - 1.45) | 1.28 (1.10 - 1.48) | 1.04 (0.96 - 1.13) | **<0.001** |

Supplemental Table II. Linear regression models evaluating the association between perfusion abnormality group and 24-hour NIHSS after individual adjustment for admission NIHSS, thrombolysis, EVT target vessel, and mTICI score.

| **Variable** | **Model 1** | | **Model 2** | | **Model 3** | | **Model 4** | |
| --- | --- | --- | --- | --- | --- | --- | --- | --- |
|  | Adjusted β (95% CI) | p-value | Adjusted β (95% CI) | p-value | Adjusted β (95% CI) | p-value | Adjusted β (95% CI) | p-value |
| Perfusion abnormality groups | | | | | | | | |
| Hyperemia | 1.72 (-1.6-5.03) | 0.306 | 1.68 (-2.38-5.73) | 0.413 | 1.81 (-2.10-5.72) | 0.359 | 1.21 (-2.99-5.41) | 0.569 |
| Hypoperfusion | 9.05 (5.39-12.70) | **<0.001** | 7.60 (3.12-12.08) | **0.001** | 7.18 (2.86-11.50) | **0.001** | 7.44 (2.66-12.23) | **0.003** |
| Both | 6.84 (2.19-11.49) | **0.004** | 6.76 (1.00-12.52) | **0.022** | 8.28 (2.53-14.03) | **0.005** | 6.67 (0.68-12.66) | **0.030** |
| Age |  |  |  |  |  |  |  |  |
| Sex (female) |  |  |  |  |  |  |  |  |
| Admission NIHSS | 0.55 (0.38-0.72) | **<0.001** |  |  |  |  |  |  |
| Preadmission mRS score |  |  |  |  |  |  |  |  |
| Thrombolysis |  |  | -2.39 (-5.74-0.95) | 0.159 |  |  |  |  |
| Admission to thrombectomy start time (min) |  |  |  |  |  |  |  |  |
| EVT target vessel | | | | | | | | |
| M1 |  |  |  |  | -4.89 (-8.74- -1.04) | **0.013** |  |  |
| M2 |  |  |  |  | -6.25 (-10.58- -1.92) | **0.005** |  |  |
| M3 |  |  |  |  | -12.20 (-26.63-2.24) | **0.096** |  |  |
| mTICI score | | | | | | | | |
| 2b |  |  |  |  |  |  | 0.93 (-2.79-4.64) | 0.621 |
| 2c |  |  |  |  |  |  | 2.81 (-1.45-7.07) | 0.194 |
